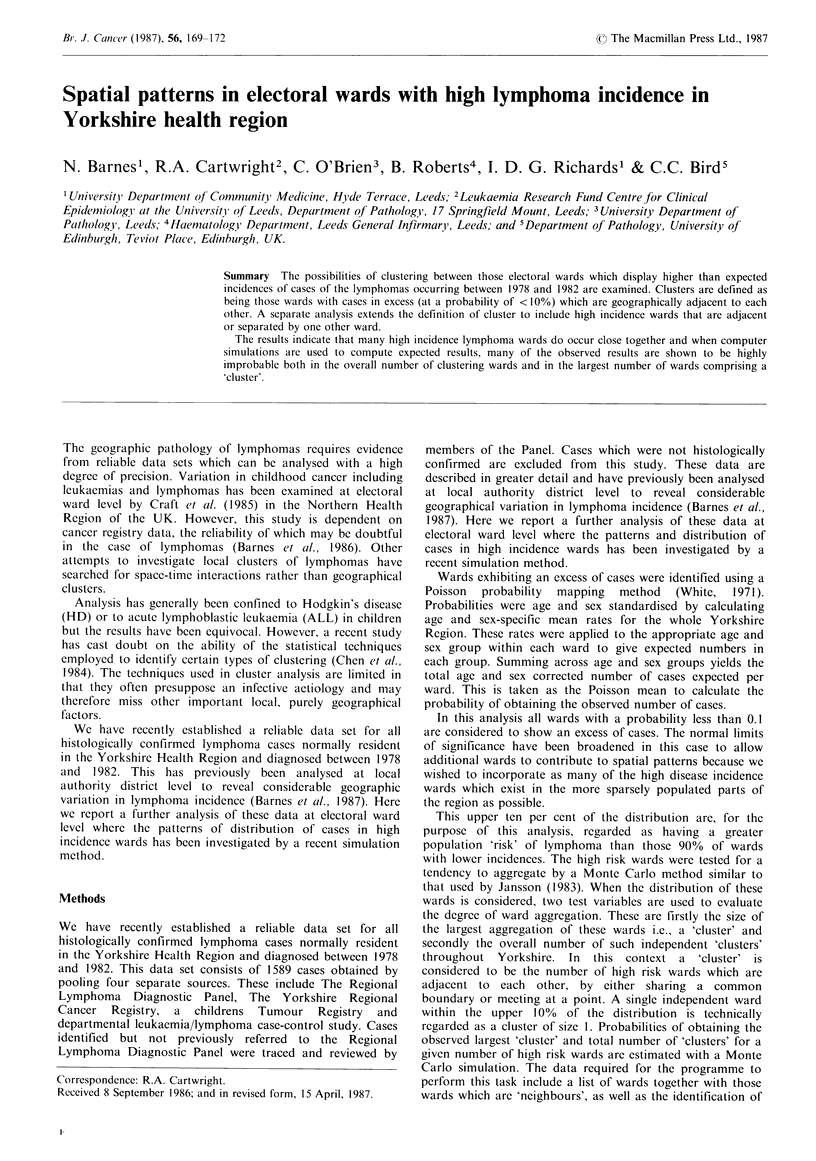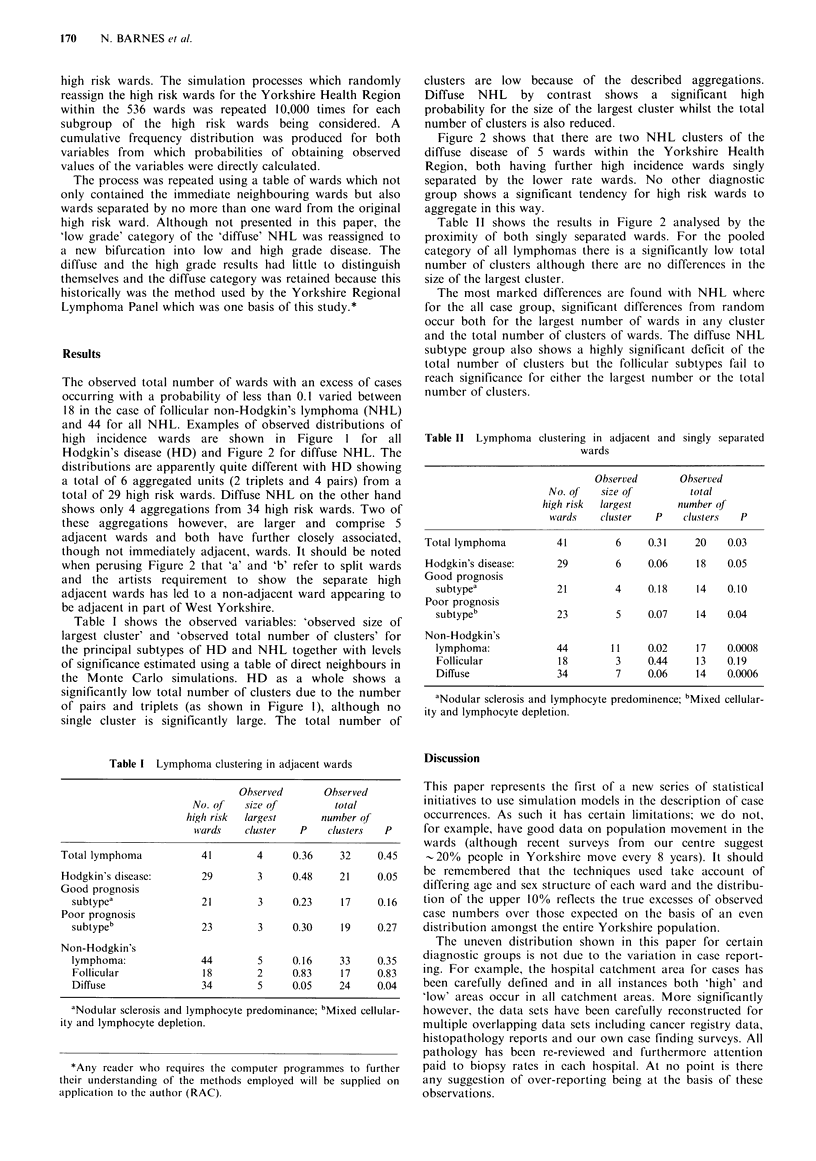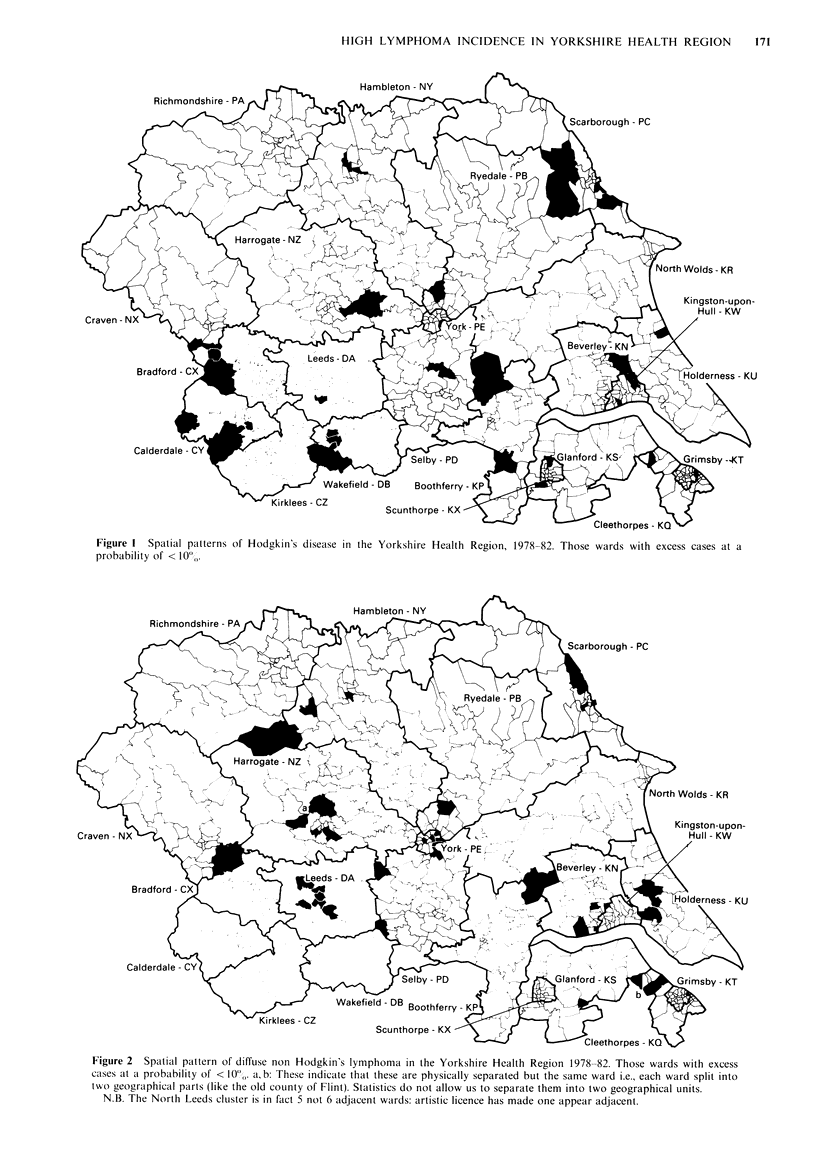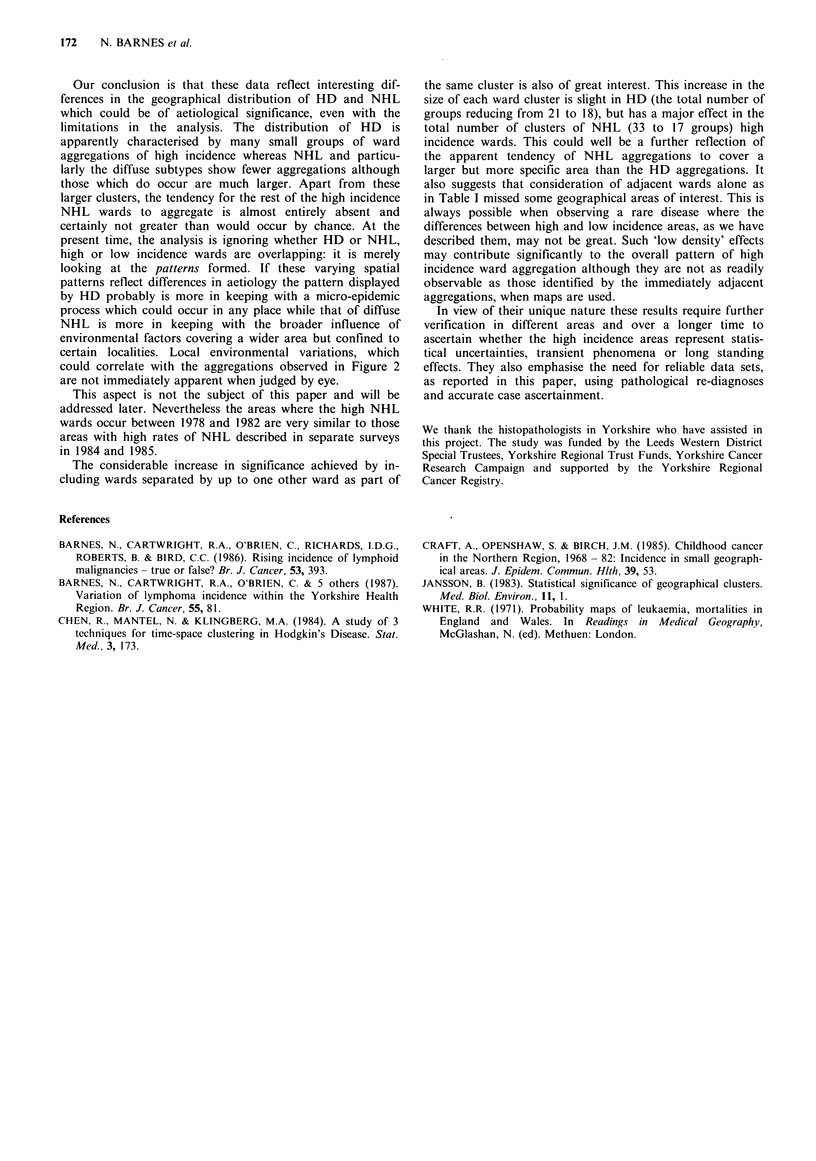# Spatial patterns in electoral wards with high lymphoma incidence in Yorkshire health region.

**DOI:** 10.1038/bjc.1987.179

**Published:** 1987-08

**Authors:** N. Barnes, R. A. Cartwright, C. O'Brien, B. Roberts, I. D. Richards, C. C. Bird

**Affiliations:** University Department of Community Medicine, Leeds.

## Abstract

The possibilities of clustering between those electoral wards which display higher than expected incidences of cases of the lymphomas occurring between 1978 and 1982 are examined. Clusters are defined as being those wards with cases in excess (at a probability of less than 10%) which are geographically adjacent to each other. A separate analysis extends the definition of cluster to include high incidence wards that are adjacent or separated by one other ward. The results indicate that many high incidence lymphoma wards do occur close together and when computer simulations are used to compute expected results, many of the observed results are shown to be highly improbable both in the overall number of clustering wards and in the largest number of wards comprising a 'cluster'.


*Br. J. Cancer* (1987), **56**, 169–172 © The Macmillan Press Ltd., 1987

### Spatial patterns in electoral wards with high lymphoma incidence in Yorkshire health region

N. Barnes[1], R.A. Cartwright[2], C. O'Brien[3], B. Roberts[4], I. D. G. Richards[1] & C.C. Bird[5]

[1]*University Department of Community Medicine, Hyde Terrace, Leeds;* [2]*Leukaemia Research Fund Centre for Clinical Epidemiology at the University of Leeds, Department of Pathology, 17 Springfield Mount, Leeds;* [3]*University Department of Pathology, Leeds;* [4]*Haematology Department, Leeds General Infirmary, Leeds; and* [5]*Department of Pathology, University of Edinburgh, Teviot Place, Edinburgh, UK.*

**Summary** The possibilities of clustering between those electoral wards which display higher than expected incidences of cases of the lymphomas occurring between 1978 and 1982 are examined. Clusters are defined as being those wards with cases in excess (at a probability of <10%) which are geographically adjacent to each other. A separate analysis extends the definition of cluster to include high incidence wards that are adjacent or separated by one other ward.

The results indicate that many high incidence lymphoma wards do occur close together and when computer simulations are used to compute expected results, many of the observed results are shown to be highly improbable both in the overall number of clustering wards and in the largest number of wards comprising a 'cluster'.

The geographic pathology of lymphomas requires evidence from reliable data sets which can be analysed with a high degree of precision. Variation in childhood cancer including leukaemias and lymphomas has been examined at electoral ward level by Craft *et al.* (1985) in the Northern Health Region of the UK. However, this study is dependent on cancer registry data, the reliability of which may be doubtful in the case of lymphomas (Barnes *et al.*, 1986). Other attempts to investigate local clusters of lymphomas have searched for space-time interactions rather than geographical clusters.

Analysis has generally been confined to Hodgkin's disease (HD) or to acute lymphoblastic leukaemia (ALL) in children but the results have been equivocal. However, a recent study has cast doubt on the ability of the statistical techniques employed to identify certain types of clustering (Chen *et al.*, 1984). The techniques used in cluster analysis are limited in that they often presuppose an infective aetiology and may therefore miss other important local, purely geographical factors.

We have recently established a reliable data set for all histologically confirmed lymphoma cases normally resident in the Yorkshire Health Region and diagnosed between 1978 and 1982. This has previously been analysed at local authority district level to reveal considerable geographic variation in lymphoma incidence (Barnes *et al.*, 1987). Here we report a further analysis of these data at electoral ward level where the patterns of distribution of cases in high incidence wards has been investigated by a recent simulation method.

### Methods

We have recently established a reliable data set for all histologically confirmed lymphoma cases normally resident in the Yorkshire Health Region and diagnosed between 1978 and 1982. This data set consists of 1589 cases obtained by pooling four separate sources. These include The Regional Lymphoma Diagnostic Panel, The Yorkshire Regional Cancer Registry, a childrens Tumour Registry and departmental leukaemia/lymphoma case-control study. Cases identified but not previously referred to the Regional Lymphoma Diagnostic Panel were traced and reviewed by members of the Panel. Cases which were not histologically confirmed are excluded from this study. These data are described in greater detail and have previously been analysed at local authority district level to reveal considerable geographical variation in lymphoma incidence (Barnes *et al.*, 1987). Here we report a further analysis of these data at electoral ward level where the patterns and distribution of cases in high incidence wards has been investigated by a recent simulation method.

Wards exhibiting an excess of cases were identified using a Poisson probability mapping method (White, 1971). Probabilities were age and sex standardised by calculating age and sex-specific mean rates for the whole Yorkshire Region. These rates were applied to the appropriate age and sex group within each ward to give expected numbers in each group. Summing across age and sex groups yields the total age and sex corrected number of cases expected per ward. This is taken as the Poisson mean to calculate the probability of obtaining the observed number of cases.

In this analysis all wards with a probability less than 0.1 are considered to show an excess of cases. The normal limits of significance have been broadened in this case to allow additional wards to contribute to spatial patterns because we wished to incorporate as many of the high disease incidence wards which exist in the more sparsely populated parts of the region as possible.

This upper ten per cent of the distribution are, for the purpose of this analysis, regarded as having a greater population 'risk' of lymphoma than those 90% of wards with lower incidences. The high risk wards were tested for a tendency to aggregate by a Monte Carlo method similar to that used by Jansson (1983). When the distribution of these wards is considered, two test variables are used to evaluate the degree of ward aggregation. These are firstly the size of the largest aggregation of these wards i.e., a 'cluster' and secondly the overall number of such independent 'clusters' throughout Yorkshire. In this context a 'cluster' is considered to be the number of high risk wards which are adjacent to each other, by either sharing a common boundary or meeting at a point. A single independent ward within the upper 10% of the distribution is technically regarded as a cluster of size 1. Probabilities of obtaining the observed largest 'cluster' and total number of 'clusters' for a given number of high risk wards are estimated with a Monte Carlo simulation. The data required for the programme to perform this task include a list of wards together with those wards which are 'neighbours', as well as the identification of

Correspondence: R.A. Cartwright.
Received 8 September 1986; and in revised form, 15 April, 1987.

high risk wards. The simulation processes which randomly reassign the high risk wards for the Yorkshire Health Region within the 536 wards was repeated 10,000 times for each subgroup of the high risk wards being considered. A cumulative frequency distribution was produced for both variables from which probabilities of obtaining observed values of the variables were directly calculated.

The process was repeated using a table of wards which not only contained the immediate neighbouring wards but also wards separated by no more than one ward from the original high risk ward. Although not presented in this paper, the 'low grade' category of the 'diffuse' NHL was reassigned to a new bifurcation into low and high grade disease. The diffuse and the high grade results had little to distinguish themselves and the diffuse category was retained because this historically was the method used by the Yorkshire Regional Lymphoma Panel which was one basis of this study.*

### Results

The observed total number of wards with an excess of cases occurring with a probability of less than 0.1 varied between 18 in the case of follicular non-Hodgkin's lymphoma (NHL) and 44 for all NHL. Examples of observed distributions of high incidence wards are shown in Figure 1 for all Hodgkin's disease (HD) and Figure 2 for diffuse NHL. The distributions are apparently quite different with HD showing a total of 6 aggregated units (2 triplets and 4 pairs) from a total of 29 high risk wards. Diffuse NHL on the other hand shows only 4 aggregations from 34 high risk wards. Two of these aggregations however, are larger and comprise 5 adjacent wards and both have further closely associated, though not immediately adjacent, wards. It should be noted when perusing Figure 2 that 'a' and 'b' refer to split wards and the artists requirement to show the separate high adjacent wards has led to a non-adjacent ward appearing to be adjacent in part of West Yorkshire.

Table I shows the observed variables: 'observed size of largest cluster' and 'observed total number of clusters' for the principal subtypes of HD and NHL together with levels of significance estimated using a table of direct neighbours in the Monte Carlo simulations. HD as a whole shows a significantly low total number of clusters due to the number of pairs and triplets (as shown in Figure 1), although no single cluster is significantly large. The total number of clusters are low because of the described aggregations. Diffuse NHL by contrast shows a significant high probability for the size of the largest cluster whilst the total number of clusters is also reduced.

Figure 2 shows that there are two NHL clusters of the diffuse disease of 5 wards within the Yorkshire Health Region, both having further high incidence wards singly separated by the lower rate wards. No other diagnostic group shows a significant tendency for high risk wards to aggregate in this way.

Table II shows the results in Figure 2 analysed by the proximity of both singly separated wards. For the pooled category of all lymphomas there is a significantly low total number of clusters although there are no differences in the size of the largest cluster.

The most marked differences are found with NHL where for the all case group, significant differences from random occur both for the largest number of wards in any cluster and the total number of clusters of wards. The diffuse NHL subtype group also shows a highly significant deficit of the total number of clusters but the follicular subtypes fail to reach significance for either the largest number or the total number of clusters.

**Table I** Lymphoma clustering in adjacent wards

| | *No. of high risk wards* | *Observed size of largest cluster* | *P* | *Observed total number of clusters* | *P* |
|---|---|---|---|---|---|
| Total lymphoma | 41 | 4 | 0.36 | 32 | 0.45 |
| Hodgkin's disease: | 29 | 3 | 0.48 | 21 | 0.05 |
| Good prognosis subtype[a] | 21 | 3 | 0.23 | 17 | 0.16 |
| Poor prognosis subtype[b] | 23 | 3 | 0.30 | 19 | 0.27 |
| Non-Hodgkin's lymphoma: | 44 | 5 | 0.16 | 33 | 0.35 |
| Follicular | 18 | 2 | 0.83 | 17 | 0.83 |
| Diffuse | 34 | 5 | 0.05 | 24 | 0.04 |

[a]Nodular sclerosis and lymphocyte predominance; [b]Mixed cellularity and lymphocyte depletion.

*Any reader who requires the computer programmes to further their understanding of the methods employed will be supplied on application to the author (RAC).

**Table II** Lymphoma clustering in adjacent and singly separated wards

| | *No. of high risk wards* | *Observed size of largest cluster* | *P* | *Observed total number of clusters* | *P* |
|---|---|---|---|---|---|
| Total lymphoma | 41 | 6 | 0.31 | 20 | 0.03 |
| Hodgkin's disease: | 29 | 6 | 0.06 | 18 | 0.05 |
| Good prognosis subtype[a] | 21 | 4 | 0.18 | 14 | 0.10 |
| Poor prognosis subtype[b] | 23 | 5 | 0.07 | 14 | 0.04 |
| Non-Hodgkin's lymphoma: | 44 | 11 | 0.02 | 17 | 0.0008 |
| Follicular | 18 | 3 | 0.44 | 13 | 0.19 |
| Diffuse | 34 | 7 | 0.06 | 14 | 0.0006 |

[a]Nodular sclerosis and lymphocyte predominence; [b]Mixed cellularity and lymphocyte depletion.

### Discussion

This paper represents the first of a new series of statistical initiatives to use simulation models in the description of case occurrences. As such it has certain limitations; we do not, for example, have good data on population movement in the wards (although recent surveys from our centre suggest ~20% people in Yorkshire move every 8 years). It should be remembered that the techniques used take account of differing age and sex structure of each ward and the distribution of the upper 10% reflects the true excesses of observed case numbers over those expected on the basis of an even distribution amongst the entire Yorkshire population.

The uneven distribution shown in this paper for certain diagnostic groups is not due to the variation in case reporting. For example, the hospital catchment area for cases has been carefully defined and in all instances both 'high' and 'low' areas occur in all catchment areas. More significantly however, the data sets have been carefully reconstructed for multiple overlapping data sets including cancer registry data, histopathology reports and our own case finding surveys. All pathology has been re-reviewed and furthermore attention paid to biopsy rates in each hospital. At no point is there any suggestion of over-reporting being at the basis of these observations.

**Figure 1** Spatial patterns of Hodgkin's disease in the Yorkshire Health Region, 1978–82. Those wards with excess cases at a probability of $<10\%$.

**Figure 2** Spatial pattern of diffuse non Hodgkin's lymphoma in the Yorkshire Health Region 1978–82. Those wards with excess cases at a probability of $<10\%$. a, b: These indicate that these are physically separated but the same ward i.e., each ward split into two geographical parts (like the old county of Flint). Statistics do not allow us to separate them into two geographical units.
N.B. The North Leeds cluster is in fact 5 not 6 adjacent wards: artistic licence has made one appear adjacent.

Our conclusion is that these data reflect interesting differences in the geographical distribution of HD and NHL which could be of aetiological significance, even with the limitations in the analysis. The distribution of HD is apparently characterised by many small groups of ward aggregations of high incidence whereas NHL and particularly the diffuse subtypes show fewer aggregations although those which do occur are much larger. Apart from these larger clusters, the tendency for the rest of the high incidence NHL wards to aggregate is almost entirely absent and certainly not greater than would occur by chance. At the present time, the analysis is ignoring whether HD or NHL, high or low incidence wards are overlapping: it is merely looking at the *patterns* formed. If these varying spatial patterns reflect differences in aetiology the pattern displayed by HD probably is more in keeping with a micro-epidemic process which could occur in any place while that of diffuse NHL is more in keeping with the broader influence of environmental factors covering a wider area but confined to certain localities. Local environmental variations, which could correlate with the aggregations observed in Figure 2 are not immediately apparent when judged by eye.

This aspect is not the subject of this paper and will be addressed later. Nevertheless the areas where the high NHL wards occur between 1978 and 1982 are very similar to those areas with high rates of NHL described in separate surveys in 1984 and 1985.

The considerable increase in significance achieved by including wards separated by up to one other ward as part of the same cluster is also of great interest. This increase in the size of each ward cluster is slight in HD (the total number of groups reducing from 21 to 18), but has a major effect in the total number of clusters of NHL (33 to 17 groups) high incidence wards. This could well be a further reflection of the apparent tendency of NHL aggregations to cover a larger but more specific area than the HD aggregations. It also suggests that consideration of adjacent wards alone as in Table I missed some geographical areas of interest. This is always possible when observing a rare disease where the differences between high and low incidence areas, as we have described them, may not be great. Such 'low density' effects may contribute significantly to the overall pattern of high incidence ward aggregation although they are not as readily observable as those identified by the immediately adjacent aggregations, when maps are used.

In view of their unique nature these results require further verification in different areas and over a longer time to ascertain whether the high incidence areas represent statistical uncertainties, transient phenomena or long standing effects. They also emphasise the need for reliable data sets, as reported in this paper, using pathological re-diagnoses and accurate case ascertainment.

We thank the histopathologists in Yorkshire who have assisted in this project. The study was funded by the Leeds Western District Special Trustees, Yorkshire Regional Trust Funds, Yorkshire Cancer Research Campaign and supported by the Yorkshire Regional Cancer Registry.

### References

BARNES, N., CARTWRIGHT, R.A., O'BRIEN, C., RICHARDS, I.D.G., ROBERTS, B. & BIRD, C.C. (1986). Rising incidence of lymphoid malignancies – true or false? *Br. J. Cancer*, **53**, 393.

BARNES, N., CARTWRIGHT, R.A., O'BRIEN, C. & 5 others (1987). Variation of lymphoma incidence within the Yorkshire Health Region. *Br. J. Cancer*, **55**, 81.

CHEN, R., MANTEL, N. & KLINGBERG, M.A. (1984). A study of 3 techniques for time-space clustering in Hodgkin's Disease. *Stat. Med.*, **3**, 173.

CRAFT, A., OPENSHAW, S. & BIRCH, J.M. (1985). Childhood cancer in the Northern Region, 1968 – 82: Incidence in small geographical areas. *J. Epidem. Commun. Hlth*, **39**, 53.

JANSSON, B. (1983). Statistical significance of geographical clusters. *Med. Biol. Environ.*, **11**, 1.

WHITE, R.R. (1971). Probability maps of leukaemia, mortalities in England and Wales. In *Readings in Medical Geography*, McGlashan, N. (ed). Methuen: London.